# Aptamers: A Review of Their Chemical Properties and Modifications for Therapeutic Application

**DOI:** 10.3390/molecules24234229

**Published:** 2019-11-21

**Authors:** Tatsuo Adachi, Yoshikazu Nakamura

**Affiliations:** 1 RIBOMIC Inc., Minato-ku, Tokyo 108-0071, Japan; 2 The institute of Medical Science, The University of Tokyo, Minato-ku, Tokyo 108-8639, Japan; nak@ims.u-tokyo.ac.jp

**Keywords:** aptamer, RNA therapeutics, chemical modifications, conformational plasticity

## Abstract

Aptamers are short, single-stranded oligonucleotides that bind to specific target molecules. The shape-forming feature of single-stranded oligonucleotides provides high affinity and excellent specificity toward targets. Hence, aptamers can be used as analogs of antibodies. In December 2004, the US Food and Drug Administration approved the first aptamer-based therapeutic, pegaptanib (Macugen), targeting vascular endothelial growth factor, for the treatment of age-related macular degeneration. Since then, however, no aptamer medication for public health has appeared. During these relatively silent years, many trials and improvements of aptamer therapeutics have been performed, opening multiple novel directions for the therapeutic application of aptamers. This review summarizes the basic characteristics of aptamers and the chemical modifications available for aptamer therapeutics.

## 1. Introduction

The idea of using single-stranded oligonucleotides as affinity molecules for various target compounds was initially proposed in 1990 [1,2]. The concept is based on the characteristic of single-stranded oligonucleotides of forming unique tertiary structures, which allow specific interactions with target molecules. Aptamers are generated by an in vitro molecular evolution method known as systematic evolution of ligands by exponential enrichment (SELEX) [3,4,5]. SELEX experiments can be conducted against a variety of target molecules or elements, such as small compounds, proteins, nanoparticles, or live cells. Hence, aptamers can be used as reagents for affinity purification as well as biosensors to replace antibodies. Moreover, an anti-vascular endothelial growth factor aptamer has been approved for the treatment of age-related macular degeneration in December 2004 [6,7].

Single-stranded RNA fold into a vast set of tertiary structures depending on their different primary structures. Therefore, RNA aptamers have a high potential to act as molecular mimics of proteins. This concept arose in our previous investigations of the translation machinery, in which we found that a polypeptide release factor encodes a tripeptide anticodon as a molecular mimic of a tRNA anticodon [8,9,10]. The structural flexibility and molecular mimicry of a single-stranded polynucleotide promoted us to develop aptamer-based modulators of biological processes, especially for therapeutic applications.

In this review, we present an overview of the structural basis of aptamers and chemical modifications of aptamers for therapeutic application.

## 2. Structural Basis for Capturing Target Proteins by Aptamers

**Figure 1:**
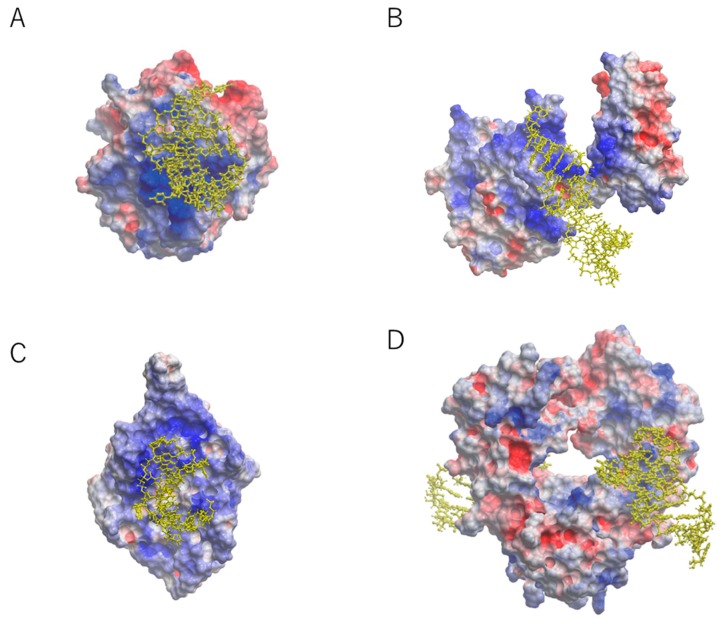
Overall structure of known aptamer–protein complexes with electrostatic surface potential [5]. The RNA aptamer is represented by a yellow ball-and-stick model. (**A**) Aptamer–thrombin complex at 1.8 Å resolution [13]. (**B**) Aptamer–nuclear factor-κB complex at 2.45 Å resolution [11]. (**C**) Aptamer–MS2 coat protein complex at 2.8 Å resolution [12]. (**D**) Aptamer–Fc region of human IgG1 (hFc1) complex at 2.15 Å resolution [16]. Blue areas: positively charged; red areas: negatively charged.

Aptamers prefer to interact with positively charged surfaces of the target proteins due to the negatively charged nature of backbone linkages. For example, the crystal structures of aptamers in complex with target proteins, such as those of nuclear factor (NF)-κB, bacteriophage MS2 capsid, and thrombin showed electrostatic interactions (Figure 1) [11,12,13]. The nucleic acid-binding domain of NF-κB and MS2 protein contribute the interaction surfaces for aptamers [11,12]. Thrombin binds to an aptamer through the positively charged surface that is naturally required for the binding of heparin [13]. Collectively, the crystal structures have indicated that electrostatic forces are among the most common sources of high affinity between aptamer and target.

We previously reported a 23-nucleotide (nt) RNA aptamer targeting the Fc domain of human IgG1 (hFc1) [14]. Since the surface of hFc1 lacks positive charges [15], this aptamer was thought to bind hFc1 through non-electrostatic forces. The aptamer showed specificity against human IgG compared with IgGs from other species [14]. The crystal structure was reported at 2.15 Å resolution (Figure 1) [16]. Unlike the electrostatic interactions mentioned above, the aptamer–protein interactions occurred at the neutral surface of hFc1 (Figure 1). This interaction seems to be the results of multiple weak forces such as hydrogen bonds and hydrophobic interactions [16]. Even though the interaction area is relatively small compared with that of other aptamer interactions, the binding between the aptamer and hFc1 is quite strong [16]. Consistent with this, the shape complementarity (SC) index of the anti-hFc1 aptamer was much greater than the SC values of 34 antibodies [17]. The fact that shape complementarity can provide considerable affinity indicates that aptamer technology is applicable not only for positively charged molecules but also for relatively neutral molecules.

## 3. Chemical Modifications of Aptamers for Therapeutic Application

**Figure 2:**
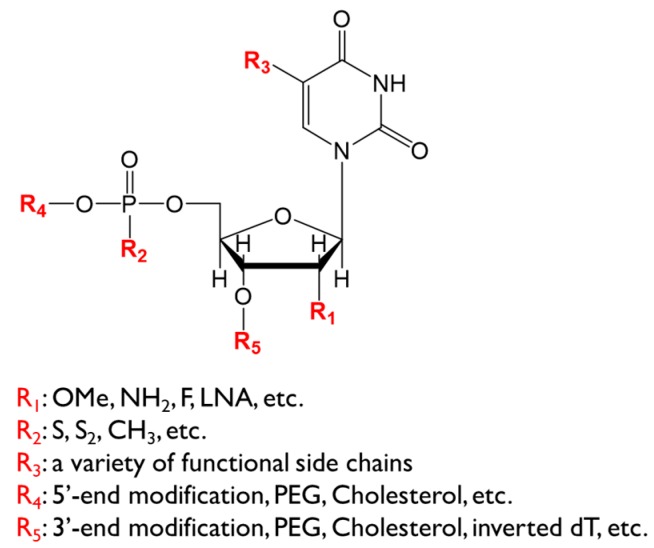
Chemical structure of a polynucleotide element. Modifications at the indicated positions are shown. OMe: O-methylation, LNA: locked nucleic acid, PEG: polyethylene glycol.

The conformational diversity and targeting specificity of RNA aptamers provide a promising potential for their therapeutic applications as inhibitors of protein–protein interactions. To achieve this goal, several features of the aptamer are required. First, therapeutic aptamers should be highly stable in the body. Since natural RNA and DNA are susceptible to endogenous nucleases, chemical modifications on the sugar and/or phosphodiester backbone should be adequately included (Figure 2). Second, the nucleotide length should be sufficiently shortened. Aptamer truncation will reduce the cost of drug manufacturing, facilitate material quality assurance, and prevent unexpected toxicity. Third, therapeutic aptamers should have good pharmacokinetics, as seen for other drugs. The following sections summarize our current understanding in the generation of therapeutic aptamers.

### 3.1. Nuclease Resistance

#### 3.1.1. Sugar Modification

Ribose modifications at the 2′ position (2′-amino, 2′-fluoro, and 2′-*O*-methyl) are widely used to confer nuclease resistance. After the identification of a candidate aptamer sequence by SELEX, these modifications could be incorporated in the course of chemical synthesis. However, introducing modifications at all nucleotides is hardly tolerated, because a sugar modification affects the aptamer activity [18]. Point-by-point modifications and activity testing are time- and cost-consuming. Therefore, an approach that integrates modified nucleotides into the SELEX process should be considered. The most important factor is the existence of a modification-permissible polymerase. The first example of ribose-modified SELEX exploited 2′-aminopyrimidines [19]. Then, mutations in the T7 RNA polymerase were explored to expand substrate compatibility. T7 RNA polymerase harboring the Y639F mutation was reported to incorporate 2′-fluoro and 2′-deoxypyrimidine [20,21,22]. Since pyrimidine modifications prevent RNase A-mediated degradation [23], this type of aptamers has been widely used as the starting point of aptamer development. However, 2′-aminopyrimidine is rarely employed in the current SELEX procedure because of its disadvantage in chemical synthesis and its negative impact on base-pairing stability in contrast to 2′-fluoropyrimidine [19,24]. Adding two more substitutions in T7 RNA polymerase, H784A and K378R, enables it to polymerize 2′-*O*-methyl RNA [25]. This 2′-*O*-methyl RNA polymerase, however, has considerably poorer incorporating activity. Therefore, priming with GMP and adding a spike level of natural NTP are required [25]. Meyer et al. screened mutations which promote 2′-*O*-methyl incorporation [26]. They found that substrate-permissible mutations at Y639 and H784 caused instability of the enzyme, which was corrected by thermostabilizing mutations [26]. The resulting enzyme had 10 mutations and could transcribe complete 2′-*O*-methyl RNA. They also pointed out that abortive products could be reduced by a promoter clearance mutation [27]. In addition to T7 RNA polymerase evolution, mutant DNA polymerase was also engineered to polymerize modified oligonucleotides. Chen and coworkers developed a 2′-*O*-methyl nucleotide polymerase from the Stoffel fragment of Taq polymerase using a phage particle-based selection system [28]. These engineered enzymes can support a more efficient generation of fully modified aptamers.

#### 3.1.2. Phosphodiester Linkage Modification

Enzymatic approaches for nuclease resistance other than sugar modifications have also been developed. Phosphorothioate and boranophosphate bonds are reported to be used in SELEX instead of the natural phosphodiester linkage [29,30]. Phosphorothioate linkage is the most common backbone modification for antisense oligonucleotide (ASO) and siRNA. Since T7 RNA polymerase accepts the S_P_ diastereomer of NTPαS [31], phosphorothioate-containing SELEX is feasible [29]. King et al. reported a selection method for a partially phosphorothioated DNA aptamer using Taq DNA polymerase [32]. In this method, dATPαS was used in place of the usual dATP [32]. Several reports followed this procedure, and Mai et al. recently developed a lymphoma-targeting aptamer through in vivo selection of a thio-dA library [33]. Although phosphorothioate linkage confers great nuclease resistance, it has the potential to cause non-specific binding towards plasma proteins compared with the normal phosphodiester bond [34]. Hence, caution will be needed when incorporating phosphorothioate into aptamers.

#### 3.1.3. Spiegelmer

NOXXON Pharma developed an alternative SELEX method for nuclease-resistant aptamers. Mirror-image oligonucleotides are not recognized by plasma nucleases [35]. Aptamers made of l-ribose-based nucleotides instead of natural d-nucleotides confer nuclease resistance and are called Spiegelmer [36,37]. Spiegelmers are selected by using mirror-image targets rather than the l-nucleotides library, which are incompatible with enzymatic manipulations. Identified d-aptamers are produced in their respective mirror-images from l-nucleotides. The resulting Spiegelmers bind, in turn, natural targets. It is worth noting that there is a disadvantage for Spiegelmers, because the availability of mirror-image targets is usually limited to relatively small-size molecules. The recent discovery of a mirror-image polymerase could improve the whole strategy for mirror-image SELEX [38,39].

#### 3.1.4. End-Capping

After selecting a sequence, nuclease resistance is further strengthened by 3′ end-capping. Inverted deoxythymidine (idT), which has a 3′–3′ linkage, is widely used to prevent 3′–5′ exonuclease activity [40]. In another way, Kasahara et al. showed that a locked nucleic acid (LNA) analogue could be attached by terminal deoxynucleotidyl transferase and provide nuclease resistance [41]. Similarly, TAGCyx Biotechnologies reported that introducing a mini-hairpin structure enhanced the nuclease resistance of aptamers [42]. These cappings do not seem to impair aptamers′ activities. Therefore, a number of aptamers are produced with end-capping.

### 3.2. Truncation

Aptamer length is another point of concern. For therapeutic application, aptamers should be shortened as much as possible. Truncation will lower the cost of material and reduce unexpected interactions. In addition, truncated seed aptamers are advantageous in aptamer discovery because of their reduced number of modifiable nucleotides for nuclease resistance. Post-SELEX truncation, however, has several difficulties. A conventional SELEX library requires a constant region at both ends of a random region for PCR amplification. These fixed sequences often form secondary structure with the random region and participate in target binding. In different cases, some aptamers require more than two distal sequences for target binding. Hence, a ubiquitous strategy for aptamer truncation is not established, and short-form-oriented SELEX would be worth considering.

One simple method for short aptamer identification is exploiting a short-nucleotides library which is composed of short random regions. Conventional libraries contain random sequence of 30–50 nt [43]. Previous reports showed successful aptamer acquisition from a random region of 15 nt [44] or 20 nt [45]. However, this concept poses a risk due to the great contribution of the fixed region. Another simple method is concealing the constant sequence. Blocking constant regions by complementary nucleotides is reported to reduce the interaction between the fixed region and the target [46].

An outstanding strategy for minimizing aptamer length is the so-called Tailored-SELEX [47,48]. This method contains two additional steps, adopter ligation and restriction, in addition to standard RNA SELEX. Briefly, random oligonucleotides flanked by a 4–6-nt fixed sequence are subjected to target binding. The selected nucleotides are ligated with primer regions and amplified by PCR. The resulting double-strand DNAs are digested by a restriction enzyme and then used in the following round. A selected aptamer should consist of a 4-nt purine leader sequence, a 30–40-nt random region, and a 3–6-nt restriction site. The adopter ligation and restriction method is also applicable to DNA SELEX [49]. These methods enable one to select aptamers that bind to a target ideally through the randomized region. The rate-limiting step is the ligation reaction. The ribose 2′-modification will further impede this process [50]. To overcome this, pre-adenylation on the 5′-end of the adopter should be considered [50]. Moreover, combining two primer-binding sites into a single adopter and circularizing would improve RT-PCR efficacy, as seen in miRNA sequence technology [51]. Although the primer-free method has the disadvantage of additional costs and time, it is a beneficial strategy if untruncatable candidates frequently arise.

### 3.3. Pharmacokinetics

Therapeutic aptamers should be stable in the body. In addition to the requirement of nuclease resistance, aptamers should also avoid systemic clearance such as renal filtration. Attachment of polyethylene glycol (PEG) is the most widely used strategy. PEGylation of aptamers decreases their glomerular filtration rate by simply increasing their molecular size. According to this notion, the SELEX procedure using a PEGylated library has been reported [52]. In this method, aptamers are transcribed with 5′-amino-GMP and then coupled with *N*-Hydroxysuccinimide (NHS)-PEG. Therefore, a PEGylated aptamer will be obtained directly through SELEX. A PEGylated library is beneficial to eliminate non-PEGylatable candidates, which share a target binding surface and the PEGylation site. In addition, PEGylated SELEX enables in vivo selection [52]. Although several PEGylated aptamers have already entered the clinical study phase, it should be noted that administration of a PEGylated aptamer can evoke anti-PEG antibodies [53]. Alternative concepts to PEGylation remain to be established.

A previous report showed that an aptamer anchored to a liposome bilayer through a lipid group had extended plasma half-life [54]. Since the estimated diameter was 50–65 nm, one expected it to evade glomerular filtration. In addition to their advantageous molecular size, serum-abundant proteins have the attractive property of a long-circulatory half-life. Human serum albumin (HSA), for example, binds to the cellular recycling neonatal Fc receptor (FcRn) and has a half-life of 19 days [55,56]. HSA has a single free thiol at position 34 (cys34). Focusing on these properties, Kuhlmann et al. elongated the serum circulation of an aptamer [57]. They utilized the cys34 residue to conjugate an oligonucleotide to HAS, and then the aptamer was hybridized on it. The resulting complex was shown to retain FcRn engagement and aptamer activity. Another plasma-enriched protein, IgG, is also recycled by FcRn and shows excellent plasma retention. A recent study showed that a coupling moiety could be introduced into the Fc fragment by an enzymatic process [58]. Therefore, IgG conjugation is a fascinating way to develop aptamers with superior pharmacokinetics.

### 3.4. Artificial Nucleotides

A number of artificial nucleotides were developed in the past decade. Artificial nucleotides have the potential to expand aptamer activity. Unnatural bases and unnatural ribose phosphate could alter the target preference of an aptamer rather than provide nuclease resistance. Permissive polymerases are again beneficial for incorporating artificial NTP into an aptamer from the early discovery stage. Aminoallyl uridine is widely used as a modified base. Aminoallyl-UTP is acceptable for T7 RNA polymerase [59]. On the basis of this compatibility, a transcript can be further modified with amine-reactive agents. SomaLogic Inc. developed another example of uridine derivatives and aptamers called SOMAmer (slow off-rate modified aptamers) [60]. They reported that a hydrophobic amino acid-like side chain enhances target binding. Moreover, the authors mentioned that the acquisition probability was greatly improved to 80% with respect to that below 30% of canonical DNA SELEX [60]. SOMAmer can be polymerized by KOD polymerase. SOMAmer showed a compact structure and exhibited a hydrophobic binding surface due to the introduced aromatic side chain [17,61]. As an example of therapeutic application, the IL-6 aptamer was developed utilizing SOMAmer [62]. Although its hydrophobicity improves the affinity, the increased number of aromatic side chain is reported to promote plasma clearance [63]. Therefore, back substitution of a natural base would be needed.

Creating new base pairs is a powerful way to augment aptamer diversity. TAGCyx Biotechnologies developed aptamers using the 7-(2-thienyl)-imidazo[4,5-b]pyridine (Ds)–2-nitro-4-propynylpyrrole (Px) base pair [64,65]. They incorporated Ds into the aptamer strand and used Px in the counter strand for PCR amplification. Deep Vent DNA polymerase can accept both artificial nucleotides. Sefah et al. reported the AEGIS (Artificially Expanded Genetic Information System)–SELEX method using six letters, i.e., GACTZP [66]. Whereas Ds–Px bases pair via shape fitting, the Z–P pair utilizes hydrogen bonds. AEGIS includes 12 nucleotides/letters from the A–T, C–G, S–B, Z–P, V–J, and K–X base pairs. Hoshika et al. showed that eight former letters, i.e., ATCGSBZP, could behave as DNA and surprisingly be transcribed into RNA by a mutant T7 RNA polymerase [67]. Hence, the use of these expanded base pairs for aptamers is quite interesting.

Unlike artificial bases, ribose-block replacement has many more limitations because of its incompatibility with enzymatic manipulation. Although 4′-thioribonucleotides are successfully incorporated by T7 RNA polymerase [68], other types of artificial ribose require a dedicated polymerase [69]. Therefore, they are not suitable for conducting many SELEX rounds. Reducing the number of rounds is necessary to overcome this inefficiency. A sophisticated method to separate aptamer–target complexes from the free nucleotide pool allows decreasing the number of selections. In some studies, capillary electrophoresis was used to integrate an LNA analogue into SELEX [70]. Further development of polymerases is needed to establish a more convenient way.

Taken together, artificial nucleotides are expected to be the next generation of aptamers. Effective polymerization method and sequence strategy must be developed. Furthermore, their biological safety must be elucidated for clinical application.

### 3.5. Computational Approach

Computational analysis has been used to assist aptamer discovery. For example, secondary structure prediction software such as MFold [71] or CentroidFold [72] are essential to estimate motif structures for target binding. In another case, the analysis of the nucleic acid pool was reported to be helpful for constructing the SELEX library [73]. It is reported that biased GC content leads to a more stem-rich aptamer pool compared to equal distribution of all four nucleotides and thus could be advantageous in the selection of structured aptamers [73]. Therefore, computer-based design would be an efficient way to optimize the initial pool or to create a patterned library.

The recent development of next-generation sequencing, also known as deep sequencing, provides vast sequence information. Hence, a bioinformatical approach for aptamer generation is worth considering. In the classical SELEX method, frequently appeared sequences after selection would be estimated as candidate aptamers. Instead of a frequency-based picking-up strategy, several algorithms for high-throughput SELEX data have been reported. MPBind (a meta-motif-based statistical framework and pipeline to predict the binding potential of SELEX-derived aptamers) exploits the Meta-Z-Score and ranking candidate sequences [74]. FASTAptamer generates information on a cluster from edit distance of each sequence [75]. FASTAptamer itself does not propose a candidate but provides cues for selection. Using this software, the TIM3 aptamer [76] and LAG3 aptamer [77] were reported. Whereas the above two programs cannot consider the secondary structure, AptaTRACE extracts important k-mers with structural information [78]. If there is an essential motif for target binding, the secondary structure profile of the specific k-mer should be enriched across the SELEX rounds. On the basis of this theory, AptaTRACE identifies a k-mer with a significant profile change by calculating the Kullback–Leibler distance between all pairs of rounds [78]. AptaTRACE, on the other hand, is neither ranking nor generating cluster information. Therefore, a ranking algorithm based on the secondary structure profile remains to be developed [79].

Collectively, in silico methods will accelerate aptamer development. Moreover, since computational procedures will reduce the selection rounds, it would be beneficial to predict an aptamer with artificial nucleotides that is difficult to make with standard SELEX. Hence, these approaches support the creation of aptamers with new chemistry.

## 4. Future Direction of Therapeutic Aptamers

The emergence of artificial nucleotides and computational methods might lead us to the next generation of aptamers. The following sections discuss the future field of therapeutic aptamers. Conventional aptamer targets are mainly focused on extracellular proteins. There is a lot of room for identifying intracellular targets. Actually, there have already been inhibitory nucleotides for transcription factors, the so-called decoy oligonucleotides. Decoy oligonucleotides are designed from the consensus sequence of DNA/RNA binding proteins and inhibit their function. Aptamers for other cellular proteins could be produced in principle. Another possible future direction is multi-functionalization of aptamers by assembling molecules. It takes advantage of the convenient chemical conjugation of aptamers.

### 4.1. Intracellular Targets

Although intracellular delivery is one of the major concerns, few strategies have been proposed. Single-stranded phosphorothioate ASO have been known to be delivered into organs without special carriers. Denichenko et al. recently developed single-stranded RNA decoy oligonucleotides targeting splicing factors [80]. The authors used the phosphorothioate backbone for an in vivo study and expected cellular internalization to occur [80]. In the case of siRNA, cholesterol conjugation was reported to enhance intracellular delivery [81,82]. In the same way, an aptamer against Hepatitis C Virus NS5B was shown to enter body organs in vivo [83]. Nanoparticle-based strategies have been explored for nucleic acids delivery. Chen et al. developed the p53 R175H aptamer [84]. In an in vivo study, they utilized nanoparticles that were originally developed for siRNA delivery [85]. NOXXON pharma reported that the HMGA1 Spiegelmer encaputured with polyethylenimine showed in vivo efficacy in a mouse xenograft model [86]. Zamay et al. developed a vimentin aptamer and attached it to the natural polysaccharide arabinogalactan [87]. The resulting complex was injected into the peritoneum and successfully inhibited adenocarcinoma growth [87].

Plasmid or viral vector-based expression is another way to implement intracellular aptamers. There are some reports that develop anti-NF-κB aptamers, which include the p50 aptamer [88,89] and the p65 aptamer [90]. Adenovirus-mediated expression of the p50 aptamer showed in vivo efficacy [91]. This aptamer-expressing strategy has the potential to be used in state-of-the-art biological technology. CRISPR (clustered regularly interspaced short palindromic repeat)-Cas (CRISPR-associated) is one of the most attractive genome-editing systems. Cas9 is localized on a specific target gene by using sgRNA (single-guide RNA). Konermann et al. reported that sgRNA has loop regions outside of Cas9, and a natural RNA aptamer could be integrated [92]. Exploiting catalytically dead Cas9 and aptamer-integrated sgRNA, they successfully regulated transcriptional activation [92]. Similar to this study, aptamer-based methods for the improvement of genome-editing accuracy [93] and for controlling latent HIV expression [94] were reported. In the future, it is reasonable to think that the use of aptamers against histone modifiers will lead to the establishment of an epigenetic regulation method on a desired locus.

### 4.2. Assembling Molecules

Generating a multi-functionalized aptamer is another interesting issue. Since an aptamer drug is usually produced by chemical synthesis, conjugating an aptamer to another molecule is easy to design. For example, aptamers engaged with chelating agents have already been reported as imaging tools in magnetic resonance imaging (MRI) [95] and positron emission tomography (PET) [96,97]. In the same way, aptamer–chelator conjugates harboring an alpha-emitting radioisotope could be used in cancer therapy.

Aptamer multimerization is an alternative way to create new functions. Tandem two-OX40 receptor aptamers activate anti-tumor immunity [98]. Since the OX40 receptor was dimerized upon endogenous ligand binding, it seems that the tandem aptamer provides a scaffold for activation [98]. Likewise, several reports showed that aptamer-mediated receptor dimerization provokes signal transduction, for instance by the Met receptor and FGFR1 [99,100]. Another study with the CD30 aptamer showed that trimerization was more effective [101]. On the other hand, it is known that some receptors are internalized and inactivated after ligand binding. Consistent with this notion, the trimer of the HER2 aptamer was reported to stimulate HER2 translocation and cause lysosomal degradation, resulting in reduced tumor growth in a xenograft model of human gastric cancer [102]. Hence, agonistic or antagonistic aptamers for therapeutic application will be developed by utilizing the multimerization method.

In addition to multivalent aptamers, connecting different aptamers is also feasible. For example, Muller et al. reported that a chimera aptamer composed of two aptamers targeting different sites of thrombin had better inhibitory activity than the monovalent version [103]. Conjugating with a receptor-binding aptamer provides an interesting characteristic. There are several reports showing that a receptor-targeting aptamer entered cells, like in the case of the HER2 aptamer. The most characterized example is the nucleolin aptamer, AS1411. Nucleolin is expressed on the cancer cell surface, and AS1411 is internalized upon binding via the micropinocytosis pathway [104] and/or clathrin-mediated endocytosis [105]. Kotula et al. reported that the β-arrestin 2 aptamer could be delivered into cells by connecting with the nucleolin aptamer [106]. Tethering the nucleolin aptamer was also reported to be an effective delivery system for siRNA [107]. Although nucleolin is selective for cancer cells, cell-internalization SELEX was reported [108], and aptamers entering other types of cells could be generated. Hence, the bifunctional strategy will be useful for the intracellular application of aptamers.

Another fascinating example of the use of bifunctional aptamers is the delivery of a therapeutic compound to the central nervous system. It is well known that the transferrin receptor (TfR)-binding molecule has the potential to cross the blood–brain barrier. The chimera aptamer consisting of the TfR aptamer and the EpCAM aptamer is reported to be delivered into the brain [109]. There are many aptamer-druggable targets for the treatment of neurodegenerative diseases, such as BACE1 [110], α-Syn [111], prion [112], and mHTT [113]. Therefore, delivering these aptamers into the brain by coupling with the TfR aptamer is an exciting research subject.

## 5. Conclusions and Perspectives

We have dedicated over one and a half decade to therapeutic aptamer discovery. Although aptamers act in a similar way to antibodies, superior characteristics such as low immunogenicity, high affinity, and chemical synthesis prompted us to develop therapeutic aptamers. Conformational plasticity of RNA is an important basis for the discovery of good therapeutic aptamers, and recent advances in SELEX technologies and chemical modifications could accelerate aptamer development. Moreover, computational approaches and artificial nucleotides described in this article further expand the possibilities to create next-generation aptamers. Once combined with the concept of functionalized aptamers, multiple successes in aptamer therapeutics could be achieved in the future.
